# Inhibition of Canonical Transient Receptor Potential Channels 4/5 with Highly Selective and Potent Small-Molecule HC-070 Alleviates Mechanical Hypersensitivity in Rat Models of Visceral and Neuropathic Pain

**DOI:** 10.3390/ijms24043350

**Published:** 2023-02-08

**Authors:** Niina Jalava, Janne Kaskinoro, Hugh Chapman, Miguel Morales, Hanna Metsänkylä, Satu-Maarit Heinonen, Ari-Pekka Koivisto

**Affiliations:** Research and Development, Orion Corporation Orion Pharma, 20360 Turku, Finland

**Keywords:** transient receptor potential channels C4/C5, TRPC4/C5 antagonist, HC-070, visceral pain, mechanical pain, neuropathic pain, colonic hypersensitivity

## Abstract

Transient receptor potential channels C4/C5 are widely expressed in the pain pathway. Here, we studied the putative analgesic efficacy of the highly selective and potent TRPC4/C5 antagonist HC-070 in rats. Inhibitory potency on human TRPC4 was assessed by using the whole-cell manual patch-clamp technique. Visceral pain sensitivity was assessed by the colonic distension test after intra-colonic trinitrobenzene sulfonic acid injection and partial restraint stress. Mechanical pain sensitivity was assessed by the paw pressure test in the chronic constriction injury (CCI) neuropathic pain model. We confirm that HC-070 is a low nanomolar antagonist. Following single oral doses (3–30 mg/kg in male or female rats), colonic hypersensitivity was significantly and dose-dependently attenuated, even fully reversed to baseline. HC-070 also had a significant anti-hypersensitivity effect in the established phase of the CCI model. HC-070 did not have an effect on the mechanical withdrawal threshold of the non-injured paw, whereas the reference compound morphine significantly increased it. Analgesic effects are observed at unbound brain concentrations near the 50% inhibitory concentration (IC_50_) recorded in vitro. This suggests that analgesic effects reported here are brought about by TRPC4/C5 blocking in vivo. The results strengthen the idea that TRPC4/C5 antagonism is a novel, safe non-opioid treatment for chronic pain.

## 1. Introduction

Global incidence of inflammatory bowel disease (IBD) is rapidly increasing, being around 300/100,000 persons. The prevalence of IBD globally exceeds 0.3%, with up to 60% of patients suffering from abdominal pain [1,2,3]. Chronic low back pain (CLBP) is the most common disease, causing suffering and disability globally [4], and with a comorbidity of severe IBD (ca. 40% of patients) [3,5]. Abdominal pain is a common symptom of IBD, i.e., Crohn’s disease and ulcerative colitis [6]. It is believed that dietary irritants, bacteria, and viruses may elicit local inflammation in the gut and sensitize chemo-receptive enterochromaffin (EC) cells, which in turn sensitize afferent nerve fibers innervating the large intestines [7,8]. Chronic stress is a risk factor and common comorbidity of both IBD and CLBP and it may sensitize peripheral and central pain circuits [6,7,9,10].

Poor efficacy and tolerability limit the chronic use of existing analgesics. Transient receptor potential (TRP) C4/C5 channels are emerging as promising non-opioid drug targets for treatment of chronic pain and other diseases [11]. In the physiological situation, TRPC4/5 channels are probably not activated to a large extent in the pain pathway. However, TRPC4/5 contribute to a wide variety of physiological processes in non-pain-sensing tissues, such as smooth muscle. TRPC4 and TRPC5 have ubiquitous expression in peripheral sensory neurons, transducing painful stimuli [12,13,14,15], in spinal cord neurons, amplifying pain signals [15], and in the brain, with an important site being the amygdala in the processing and control of the affective-emotional dimension, i.e., unpleasantness of pain [16,17,18,19,20,21,22,23]. Several agonists, receptors, and stimuli, such as lysophosphatidylcholine (LPC) species, diacylglycerol (DAG), G protein-coupled receptors (GPCRs), cold, and membrane stretch, activate TRPC4/C5 channels, resulting in increased depolarization of target cells such as neurons [13,20,21,24,25,26,27]. Taken together, the expression and function suggest that TRPC4/C5 channels may facilitate pain and that inhibition of such channels seems a plausible mechanism to alleviate pain.

Although several structurally different small-molecule TRPC4/C5 inhibitors have been described [28], the lack of highly selective and potent compounds together with limited data on pharmacokinetics has complicated their use in in vivo studies. ML-204 was described as the first micromolar blocker of TRPC4/C5 channels [29]. Intraperitoneal, and oral to a lesser extent, ML-204 administration dose-dependently attenuated intracolonic mustard oil-induced visceral pain-related behaviors and hindpaw mechanical hypersensitivity [30]. Amygdaloid ML-204 application produces a dose-dependent suppression of mechanical hypersensitivity, and affective-like spontaneous pain-related behavior in a rat spared nerve injury model of neuropathic pain [22]. However, ML-204 may also interact with muscarinic receptors [31]. Furthermore, hindpaw injection of the micromolar TRPC5 small-molecule inhibitor AC1903 [32] prevents the persistent mechanical pain in mouse hindpaw plantar incision and complete Freund’s adjuvant (CFA) models of inflammatory pain, as well as in a model of neuropathic pain induced by chemotherapy treatment with paclitaxel [13]. On the other hand, AC1903 was recently shown to interact with several TRP channels and other proteins, a result which suggests that in vivo results obtained with AC1903 cannot be explained solely through TRPC4/C5 inhibition [33].

Here, we test the hypothesis of whether systemic exposure of the highly selective and potent TRPC4/C5 inhibitor HC-070 [27] is able to provide analgesia. We studied the effects of a single oral HC-070 treatment on colonic hypersensitivity in male/female rat visceral pain by using the model of peripheral colorectal sensitization induced by trinitrobenzene sulfonic acid (TNBS) [34] and the model of partial restraint stress (PRS) [35,36] to assess whether stress can sensitize colonic nociceptors and central pain processing circuits. In both models, HC-070 treatment dose-dependently reduced visceral pain-associated colonic hypersensitivity, assessed with the colonic distension test. In the male rat neuropathic pain model of chronic constriction injury, HC-070 after a single dose was found to alleviate mechanical hypersensitivity in the paw pressure test without impacting the mechanical sensitivity of the non-injured control paw. Pharmacokinetics was assessed for HC-070 plasma and brain exposures at different time points. HC-070 plasma and brain protein binding was studied by using the rapid equilibrium dialysis method. Evaluation of the pharmacokinetic (PK)–pharmacodynamic (PD) relationship showed that the analgesic efficacy of HC-070 coincides with unbound brain exposures near the 50% inhibitory concentration (IC_50_) for TRPC4. These data with HC-070 suggest that the observed anti-hypersensitivity effects are on target and that TRPC4/C5 channels are promising and safe targets for the treatment of visceral and neuropathic pain.

## 2. Results

### 2.1. Assessment of Primary Pharmacology of HC-070 Using Manual Patch-Clamp

**Figure 1:**
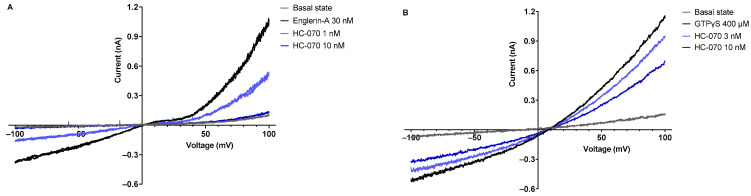
Assessment of primary pharmacology of HC-070 using manual patch-clamp. hTRPC4 current–voltage relationships with activation by Englerin A (**A**) and GTPγS (**B**).

In patch-clamp recordings, HC-070 exhibited concentration-dependent inhibition of human TRPC4 current induced by either Englerin A or GTPγS. The IC_50_ values of HC-070 were 0.96 nM with Englerin A (n = 4) (Figure 1A) and 5.72 nM with GTPγS (n = 3) (Figure 1B) at a membrane potential of −100 mV.

### 2.2. Anti-Hypersensitivity Effect of TRPC4/5 Antagonist HC-070 in the Model of Trinitrobenzene Sulfonic Acid-Induced Colonic Hypersensitivity in Male Rats

**Figure 2:**
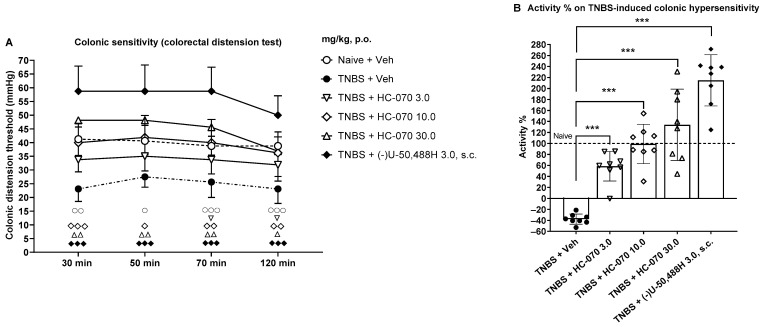
Effect of a single HC-070 (mg/kg, p.o.) administration on hypersensitivity in the rat model of trinitrobenzene sulfonic acid (TNBS)-induced chronic colonic hypersensitivity. (**A**) The colorectal distension test was performed 30 min, 50 min, 70 min, and 120 min after HC-070/Vehicle/(−)U-50,488H treatment. Results of colonic distension threshold (mmHg) evoked by the colorectal distension test are expressed as mean, the error bars represent SD, n = 8/treatment group. One symbol indicates treatment *p* < 0.05, two symbols *p* < 0.01, and three symbols *p* < 0.001, as compared to the vehicle-treated TNBS group, Dunnett’s test after two-way repeated measures ANOVA or Dunn’s test after the Kruskal–Wallis test. (**B**) Results of percentual colonic anti-hypersensitivity activities of HC-070 or percentual variation of TBNS + Veh vs. naive animals are expressed as mean of four trials, the error bars represent SD, n = 8/treatment group, and each symbol represents an individual data point. ***, *p* < 0.001, as compared to the vehicle-treated TNBS group, Dunnett’s test after significant one-way ANOVA.

Treatment had a significant main effect on the colonic distension threshold (mmHg), evoked by gradually increased intra-colonic pressure to induce a pain-related behavioral response, measured at 30, 50, 70, and 120 min (F5,42 = 26.76; *p* < 0.0001; Figure 2A). Trinitrobenzene sulfonic acid (TNBS) induced statistically significant colonic hypersensitivity, assessed seven days after injection (*p* < 0.01 at 30 min, *p* < 0.05 at 50 min, and *p* < 0.001 at 70 and 120 min, post hoc test between vehicle-treated naïve and TNBS animals; Figure 2A). The naïve colonic distension threshold was in the range of 39–41 mmHg, while the TNBS injection decreased the colonic distension threshold to a level of 23–28 mmHg. The mean percentual variation of four consecutive colonic distension trials due to TNBS administration as compared to naïve rats was −38% (Figure 2B). Moreover, post hoc testing indicated that colonic hypersensitivity induced by TNBS was significantly and in a dose-dependent manner attenuated by HC-070 3 mg/kg at 70 and 120 min, as well as reversed by 10 mg/kg and 30 mg/kg at all the studied time points post-dosing (Figure 2A). The mean percentual colonic anti-hypersensitivity activities of four consecutive colonic distension trials were 58%, 99%, and 134% for 3, 10, and 30 mg/kg, respectively (Figure 2B). Additionally, the subcutaneously administered κ-opioid agonist (−)U-50,488H, 3 mg/kg, induced significant reversal of the lowered visceral pain threshold in response to colonic distension due to the TNBS injection (Figure 2A). The corresponding mean percentual colonic anti-hypersensitivity activity of four consecutive colonic distension trials was 215% for (−)U-50,488H (Figure 2B).

### 2.3. Colonic Anti-Hypersensitivity Effect of TRPC4/5 Antagonist HC-070 in the Model of Partial Restraint Stress in Female Rats

**Figure 3:**
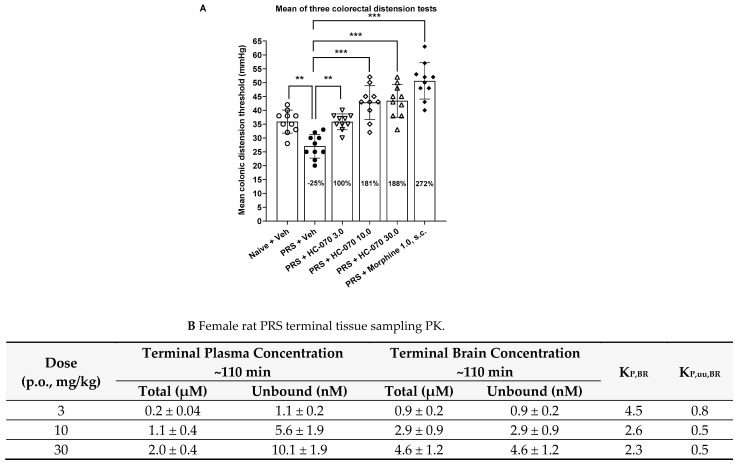
Effect of a single administration of HC-070 (mg/kg, p.o.) on colonic hypersensitivity in the female rat model of partial restraint stress (PRS). (**A**) Results of the colonic distension threshold (mmHg) evoked by the colorectal distension test are expressed as the mean of three trials, the error bars represent SD, n = 10/treatment group, and each symbol represents an individual data point. Percentages are expressed as colonic anti-hypersensitivity activity of HC-070 and morphine, or as percentage of variation as compared to naive animals. **, ***: *p* < 0.01 and *p* < 0.001, respectively, as compared to the vehicle-treated PRS group, Dunnett’s test after significant one-way ANOVA. (**B**) Average exposures of terminal plasma and brain at the end of the study from female rats dosed orally with 3, 10, or 30 mg/kg of HC-070. Error bars show standard deviation. Unbound fraction in rat plasma was 0.5% (0.005), and 0.1% (0.001) in rat brain.

Results of the colonic distension threshold (mmHg), evoked by gradually increased intra-colonic pressure to induce a pain-related behavioral response, are expressed as the mean of three trials. Partial restraint stress (PRS) induced statistically significant colonic hypersensitivity (F5,54 = 25.12, *p* < 0.0001; *p* < 0.0014, post hoc test between PRS and naïve groups; Figure 3A). The naïve colonic distension threshold was in the range of 36 mmHg, while partial restraint stress decreased the colonic distension threshold to a level of 27 mmHg. The percentual variation due to partial restraint stress as compared to naïve rats was −25% (Figure 3A). Furthermore, post hoc testing indicated that normal colonic sensibility was dose-dependently restored after oral HC-070 doses of 3, 10, and 30 mg/kg by percentual anti-hypersensitivity activities at a colonic hypersensitivity of 100%, 181%, and 188%, respectively. Additionally, subcutaneously administered morphine at 1 mg/kg induced significant reversal of the lowered visceral pain threshold in response to colonic distension. The corresponding percentual anti-hypersensitivity activity on the colonic hypersensitivity of morphine was 272% (Figure 3A).

### 2.4. Mechanical Anti-Hypersensitivity Effect of TRPC4/5 Antagonist HC-070 in the Model of Chronic Constriction Injury in Male Rats

**Figure 4:**
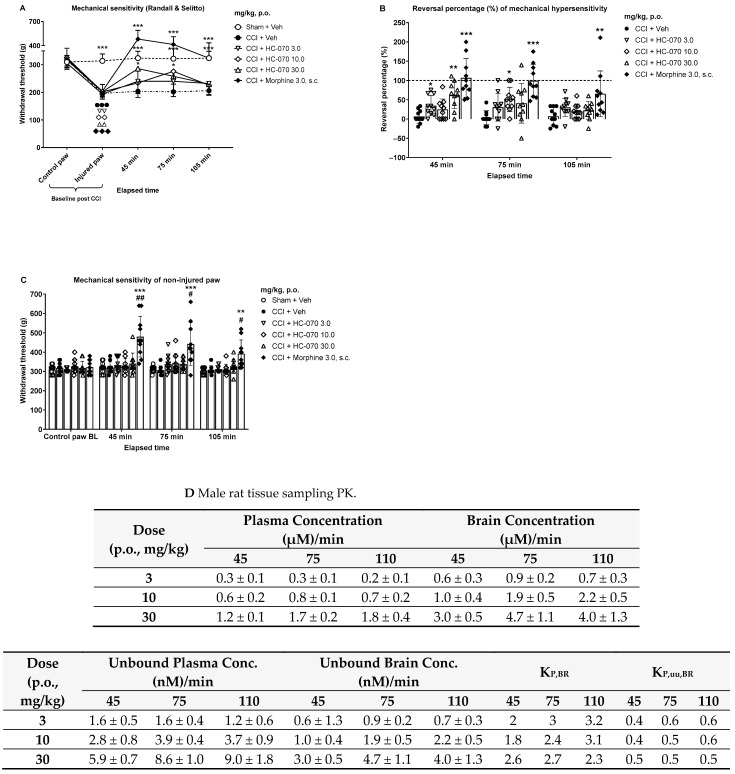
Effect of a single administration of HC-070 (mg/kg, p.o.) on injured paw mechanical hypersensitivity in the rat model of peripheral neuropathic pain (chronic constriction injury, CCI). (**A**) Results of the withdrawal threshold (g) evoked by the paw pressure test are expressed as the mean, the error bars represent SD, n = 10/treatment group. Two symbols indicates treatment *p* < 0.01, three symbols *p* < 0.001, as compared to the control paw of the corresponding group, Dunnett’s test after significant two-way ANOVA or the Wilcoxon matched-pairs signed rank test. * *p* < 0.05, *** *p* < 0.001, as compared to the vehicle-treated CCI group, Dunnett’s test after significant two-way repeated measures ANOVA or Dunn’s test after a significant Kruskal–Wallis test. (**B**) Results of reversal percentage (%) are expressed as the mean, the error bars represent SD, n = 10/treatment group, and each symbol represents an individual data point. Dashed line illustrates the level of full reversal on CCI-induced mechanical hypersensitivity. *, **, ***: *p* < 0.05, *p* < 0.01, *p* < 0.001, respectively, as compared to the vehicle-treated CCI group, Dunnett’s test after significant two-way repeated measures ANOVA or Dunn’s test after a significant Kruskal–Wallis test. (**C**) Results of the withdrawal threshold (g) evoked by the paw pressure test are expressed as the mean, the error bars represent SD, n = 10/treatment group, and each symbol represents an individual data point. #, ##: *p* < 0.05 and *p* < 0.01, respectively, as compared to the baseline (BL) of the corresponding group, Dunnett’s test after significant two-way ANOVA. **, ***: *p* < 0.01, *p* < 0.001, respectively, as compared to the vehicle-treated CCI group, Dunn’s test after significant Kruskal–Wallis test. (**D**) Average plasma and brain exposures from rats dosed orally with 3, 10, or 30 mg/kg of HC-070, 45, 75, and 110 min post-dosing. Error bars show the standard deviation. Unbound fraction in rat plasma was 0.5% (0.005), and 0.1% (0.001) in rat brain.

Sciatic nerve ligation, a chronic constriction injury (CCI), induced statistically significant mechanical hypersensitivity in the operated right hindpaw two weeks after the surgery (interaction between time and treatment: F20,216 = 14.59, *p* < 0.0001; *p* < 0.001, post hoc test between CCI operated and sham groups; Figure 4A). The non-injured left control paw withdrawal threshold was in the range of 306–326 g, while nerve injury decreased the ipsilateral paw withdrawal threshold to a level of 194–204 g (*p* < 0.01 or *p* < 0.001, as compared within each CCI operated group; Figure 4A). During the established phase (fourteen days after surgery), orally administered HC-070 and subcutaneous morphine administration had a significant main effect on mechanical hypersensitivity induced by CCI (*p* < 0.0001). Post hoc testing indicated that mechanical hypersensitivity induced by CCI in the late phase was significantly attenuated by HC-070 at 30 mg/kg and 10 mg/kg at 45 and 75 min post-dosing, respectively. Additionally, morphine at 3 mg/kg at 45, 75, and 105 min post-administration induced a significant anti-hypersensitivity effect in CCI operated animals (Figure 4A). Moreover, these studied compounds had a significant effect on percentual reversal of CCI-induced mechanical hypersensitivity in the injured paw during the established neuropathic phase (F4,45 = 18.20, *p* < 0.001, *p* = 0.0143 at 105 min post-dosing; Figure 3B). Interestingly, the lowest studied HC-070 dose of 3 mg/kg 45 min after dosing also showed significant anti-hypersensitivity of 34% in the injured paw, whereas the corresponding reversal percentage for 30 mg/kg at 45 min was 62% and for 10 mg/kg at 75 min was 51%. Sustained mechanical hypersensitivity induced by CCI was abolished by morphine treatment at the first two time points (Figure 4B).

HC-070 and morphine were also tested to assess the withdrawal threshold evoked by the paw pressure test in the non-injured paw. HC-070 did not have an effect on the mechanical sensitivity of the control paw. However, post hoc testing revealed that morphine treatment statistically significantly increased the mechanical withdrawal threshold (Figure 4C).

### 2.5. HC-070 Pharmacokinetics in Rat Indicates Ready Access to the Brain

Plasma and brain concentrations of HC-070 were analyzed 45 and 75 min after oral dosing of 3, 10, and 30 mg/kg in 0.5% methyl cellulose (MC) suspension to male satellite rats in connection with the chronic constriction injury (CCI) study. Furthermore, terminal plasma and brain concentrations of HC-070 were analyzed at the end of the paw pressure test in connection with the CCI study, and from female rats at the end of the last colonic distension test in the partial restraint stress study. PK evaluation confirmed that HC-070 is a centrally penetrating compound in rats. HC-070 plasma protein and brain homogenate binding data show high plasma protein binding of 99.5%, and an unbound fraction of 0.001 in rat brain homogenate. Total and unbound plasma and brain tissue concentration analyses together with the KP,uu,BR (partitioning coefficient between the unbound brain and plasma exposures) of 0.5–0.8 in female rats (Figure 3B) or 0.4–0.6 in male rats, independent of dose or sampling time point (Figure 4D), confirmed that HC-070 has ready access to the brain. The pharmacokinetic–pharmacodynamic relationship showed that pharmacologically relevant unbound exposures, similar to or lower than the 50% inhibitory concentration (IC_50_) recorded in vitro for TRPC4, were sufficient for significant efficacy in rat neuropathic and visceral pain models.

## 3. Discussion

Our study showed that acute TRPC4/C5 inhibition with small-molecule compound HC-070 alleviates, or even fully blocks, mechanical hypersensitivity associated with visceral and neuropathic pain in rats. To our knowledge, this is the first study reporting the quantitative in vitro–in vivo correlation of analgesic efficacy provided by TRPC4/C5 channel blockers.

The anti-hypersensitivity effect of HC-070 in the present study is in line with previous results with structurally different micromolar TRPC4/C5 inhibitors, ML-204 and AC1903, demonstrating that TRPC4 and TRPC5 channels are involved in pain signaling in various experimental models [7,13,22,30]. However, this study extends earlier findings by providing pharmacokinetic data in parallel with pharmacodynamics in rat pain models. Unlike ML-204 or AC1903, HC-070 is reported to be highly selective and a nanomolar inhibitor for murine and human TRPC4 and TRPC5, compared to a wide variety of ion channels, receptors, enzymes, kinases, and transporters [27], thus making it a most reliable tool compound for studying the in vivo function of these channels for now.

TRPC4 and TRPC5 channels were initially cloned and expressed as homomeric functional ion channels in heterologous cell models. It was soon found that homomeric TRPC4 and TRPC5 can form functional channels, in contrast to TRPC1 [37]. A recent study established that central TRPC1/C4/C5 channels co-assemble into a functional heterotetrameric channel and macromolecular complexes consisting of several other proteins in the brain [38]. This finding suggests that small-molecule inhibitors of TRPC4 and TRPC5 channels crossing the blood–brain barrier (BBB) are likely to block heterotetrameric complexes and do not show selectivity over TRPC4 or TRPC5 subtypes in vivo. We believe that HC-070 blocks heterotetrameric channels in vivo with similar potency to that found on homomeric TRPC4 in vitro with a 50% inhibitory concentration (IC50) = 1–6 nM, in agreement with Just et al. [27]. mRNA of TRPC1/C4/C5 channels is expressed in most peripheral sensory neuron subtypes [14,39]. Further, a recent study reported TRPC5 expression in 75% of human DRG neurons, most being putative nociceptors [13]. Interestingly, TRPC5 can be activated by membrane stretch [26,40], a mechanism that may contribute to acute mechanical pain sensing and chronic pain sensitization in vivo. How TRPC1/C4/C5 channels are assembled in the peripheral nervous system is currently not known. It remains to be seen if TRPC4 or TRPC5 selective antibodies will be developed and whether they display subtype-selective analgesic effects in vivo.

The present study demonstrated the analgesic efficacy of oral HC-070 in experimental models of visceral pain, in line with the previous report showing ML-204 efficacy in visceral pain-related behaviors and hindpaw mechanical hypersensitivity, evoked by intracolonic mustard oil [30]. Here, a single oral HC-070 dose resulted in a decrease of or even restored sustained non-inflammatory colonic hypersensitivity induced by trinitrobenzene sulfonic acid (TNBS). The colon injection of TNBS induced peripheral colorectal sensitization, mimicking certain characteristics of irritable bowel syndrome (IBS), and is considered to model pathophysiological aspects of it [34]. Furthermore, HC-070 also completely suppressed the decrease in the pain threshold to colonic distension induced by partial restraint stress (PRS) in female rats. Restraint stress, as a mild, non-painful, mainly psychological stressor in rats [36], is thought to model psychological distress associated with the onset or exacerbation of symptoms in IBS patients [41]. Further, earlier studies suggest that psychological stress as a risk factor may have a role in the enhanced pelvic nerve fiber mechanical sensitivity of healthy volunteers and IBS patients to colonic distention [8,42,43,44,45], which was mimicked here with the rat colonic distension test. Interestingly, stress response-associated norepinephrine (NE) treatment on gut epithelial enterochromaffin cells was shown to sensitize afferent colonic nerve fibers to mechanical stimulus in intestinal organoids [7]. ML-204 blocked the colonic afferents mechanical hypersensitivity induced by NE, indicating that the α2 adrenoreceptor-TRPC4 signal transduction cascade plays a role in visceral pain [7]. Moreover, along with TRPC1/C4/C5 channels being expressed by enteric neurons and smooth-muscle cells in the visceral tract [46], a more recent study identified mRNA of TRPC1/C4/C5 channels in a vast majority of colonic sensory neuron subtypes [47]. In addition, this study showed that acute oral administration of HC-070 significantly attenuated prolonged static mechanical pain hypersensitivity induced by unilateral chronic constriction injury, that displays similarities to the features of post-traumatic/postsurgical neuropathic pain and low back pain experienced by patients [48,49].

Our results showed that efficacy of HC-070 in rat visceral and neuropathic pain models was observed at free unbound brain concentrations near the in vitro-recorded IC_50_ for TRPC4, in line with Just et al.’s results regarding the anxiolytic and antidepressant effects of HC-070 in mice [27]. In addition, ML-204 administration to the amygdala had analgesic action in a spared nerve injury (SNI) model of neuropathic pain in rats [22], whereas the intra-plantar AC1903 injection failed to attenuate mechanical hypersensitivity in a mouse SNI model [13]. These results suggest that central blocking of TRPC4/C5 channels is sufficient for pain relief. Here, the in vivo analgesic efficacy of HC-070 is likely due to blockage of both peripheral and central TRPC4/C5 channels. Indeed, peripheral administration of AC1903 was demonstrated to prevent the established mechanical hypersensitivity in several different mouse models, such as intra-plantar CFA injections, hindpaw incision, and chemotherapy treatment with paclitaxel. Similar to AC1903, established postoperative mechanical hypersensitivity in incised mice was alleviated by HC-070 hindpaw administration [13]. These collective data support the idea that peripheral drive and centrally mediated TRPC4/C5 pronociceptive actions may differ between disease conditions. However, the most robust analgesic efficacy is likely achieved when both central and peripheral channels are blocked. Future studies are needed to clarify whether peripheral blocking of TRPC4/C5 channels is sufficient to bring about analgesia.

The subcutaneously administered kappa-opioid agonist (−)U-50,488H and the mu-opioid agonist morphine [34,50,51] presented potent activity on TNBS- or PRS-induced colonic hypersensitivity, respectively, but increased the colonic pain threshold well above normal, in parallel with findings in an earlier study [34]. Further, in the non-operated control paw, morphine significantly increased the mechanical withdrawal threshold, whereas HC-070 did not. These results confound interpretation of the results obtained with opioids in behavioral assays and might at least partly reflect their known unwanted effects, such as sedation, nausea, and reduced intestinal motility. Similar to HC-070 treatment here, peripheral dosing of AC1903 did not change the naïve mechanical sensitivity in uninjured mice [13] nor did oral HC-070 treatment alter the locomotor activity in mice [27]. In view of these results, TRPC4/C5 blockers appear to provide similar analgesia effects to opioids but with better tolerability and safety.

There is earlier evidence that the amygdala, highly expressing TRPC4 and TRPC5, is essential in fear, anxiety, and depression [20,21,27]. In imaging and anatomical studies, brain activity-associated neuroplastic changes in the amygdala are also reported in human low back pain and chronic neuropathic pain in rodents [16,18,52,53,54]. Interestingly, restraint stress has been shown to enhance the expression of the neural activity marker c-fos in the amygdala of rats [55,56], and G protein-coupled receptors activating TRPC5 channels in the amygdala, for example, are linked to visceral pain induction [57]. Thus, we hypothesized that TRPC4/C5 channel blocking in amygdaloid neurons encoding pain unpleasantness may partly underlie the observed analgesic efficacy of HC-070.

Chronic use of non-steroidal anti-inflammatory drugs is well-known to cause gut barrier dysfunction [58]. Nav1.8-expressing sensory neurons were recently shown to regulate goblet cell mucus production and gut barrier function [59]. Peroral and chronic dosing of TRPC4/C5 blockers is likely to achieve and maintain high exposure in the gut. It remains to be seen if chronic pharmacological blocking of TRPC4/C5 channels is better tolerated than existing medicines used for treatment of chronic pain.

Neural cell adhesion molecule (NCAM1) has been genetically associated with IBD in a genome-wide analysis of 53,400 people [5]. Back pain and limb pain were frequent comorbid diseases of IBD patients in the same study. Interestingly, NCAM1 was shown to selectively interact with TRPC4/C5 ion channels, and the NCAM1 antibody was able to open TRPC4/C5 channels in neurons, an effect that could be blocked by selective TRPC4/C5 inhibitors [60]. Taken together, these data provide compelling human genetic and functional support for the idea that the NCAM1-TRPC4/C5 pathway contributes to human pain and furthermore increases the likelihood of clinical success by at least 2-fold [61]. Our results are in full agreement that pharmacological blocking of TRPC4/C5 channels has promising potential to treat visceral and neuropathic pain.

## 4. Materials and Methods

### 4.1. Patch-Clamp Recordings to Assess HC-070 Potency on Human TRPC4

The patch-clamp technique is the gold standard in the assessment of pharmacological effects of ion channel blockers. The IC_50_ determined in patch-clamp studies is a direct measure of TRPC4/C5 channel blocking. A T-Rex-293-hTRPC4β cell line, purchased from SB Drug Discovery (Glasgow, UK), was cultured in DMEM supplemented with TET-FBS (10%), L-glutamine (2 mM), geneticin (2 mg/mL), blasticidin (5 µg/mL), and penicillin-streptomycin (100 U/mL) at 37 °C in a humdified atmosphere of 5% CO_2_. Cells were subcultured twice a week using TrypLE Express. One day prior to electrophysiological measurements, cells were incubated with media containing tetracycline (1 µg/mL).

Human TPRC4 currents were recorded with the whole-cell manual patch-clamp technique at room temperature using an Axopatch 200B amplifier and pClamp 9.2 software (Molecular Devices, San Jose, CA, USA). Drops of cell suspension were pipetted into the recording chamber of a Dynaflow Resolve chip (Fluicell, Sweden), and a cell was patched and then moved to the outflow of a channel-perfusing extracellular solution consisting of (in mM): 143 NaCl, 4 KCl, 1.8 CaCl_2_, 1.2 MgCl_2_, 5 glucose, and 10 HEPES (pH 7.4 with NaOH; osmolarity 301 ± 3 mOsm). Patch pipettes were pulled from borosilicate glass and filled with a solution consisting of (in mM): 145 CsCl, 2 MgCl_2_, 10 HEPES, 1 EGTA, 5 Na_2_ATP, and 0.1 Na_2_GTP (pH 7.2 with CsOH; osmolarity 285 ± 2 mOsm). Series resistance (by 75%) and capacitance were compensated. From a holding potential of 0 mV, a voltage ramp from –100 mV to 100 mV was repeated every 2 s. Recordings were filtered at 2 kHz and sampled at 10 kHz. Englerin A at 30 nM or GTPγS at 400 µM were used to activate hTRPC4. Cells were sequentially exposed to HC-070 concentrations from 1 to 30 nM or from 3 to 100 nM, respectively, when Englerin A or GTPγS was used.

### 4.2. Experimental Animals

The animal ethical committee (Comité d’Ethique pour l’Expérimentation Animale Auvergne–C2E2A) approved and the French Ministry of Education and Research (MESR) accredited (national authorisation number #18129 and #01101.02) the standard protocols describing the animal models used here. Animals were treated according to the guidelines of the Committee for Research and Ethical Issue of the I.A.S.P. [62] and the European guidelines 2010/63/UE. The total number of rats used was 192. We wanted to reduce the number of animals based on 3R principles, but as females are generally more prone to develop chronic (visceral) pain than males and shown to be more prone to develop stress-induced pain, both genders were used in visceral pain studies. Furthermore, the FDA also recommends the use of both sexes in preclinical studies. Drug treatment conditions and testing were performed in a random order by a blinded experimenter. For the chronic constriction injury (CCI) test, male Sprague-Dawley rats (SPF status, Janvier, France) weighing 100–140 g at the time of surgery and 220–280 g on the testing day were used. For the tissue collection to assess effective exposure levels, additional satellite male Sprague-Dawley rats (SPF status, Janvier, France) weighing 220–280 g were used. Male Sprague-Dawley rats (SPF status, Janvier, France), weighing 390–450 g on the day of surgery, were used for the colonic distension test in the model of trinitrobenzene sulfonic acid (TNBS)-induced colonic hypersensitivity. Female Wistar rats (SPF status, Janvier, France), weighing 175–215 g during the experimental phase, were used for the colonic distension test in the model of partial restraint stress (PRS). Rats were housed in a temperature (20–24 °C) and relative humidity controlled (45–65%) room. Furthermore, they were acclimatized to an artificial day/night cycle of 12 h light (6.30 a.m. to 6.30 p.m.)/12 h darkness. Animals had free access to tap water and were fed ad libitum with a pelleted complete diet (reference A04, S.A.F.E.). Two to four animals were housed per cage and acclimatized for a period of at least five days before any testing. Animals for the behavioral testing (pharmacodynamic, PD) and for the assessment of tissue exposures (pharmacokinetic, PK) were dosed in parallel.

### 4.3. Assessment of Colonic Hypersensitivity in the Rat Model of Trinitrobenzene Sulfonic Acid-Induced Colonic Hypersensitivity

Rats were fasted overnight and then anesthetized (Xylazine 10 mg/kg/Ketamine 60 mg/kg) for the surgical administration of trinitrobenzene sulfonic acid (TNBS, 50 mg/kg) into the proximal colon (1 cm from the caecum) to induce chronic hypersensitivity in the distal non-inflamed colon. After surgery, animals were returned to their homecages until pharmacological treatments seven days later. Naïve animals without surgery were placed in the same housing conditions. Vehicle (0.5% methylcellulose) or HC-070 were orally administered at 3 mL/kg, (−)U-50,488H was subcutaneously administered at 5 mL/kg, and 4 consecutive colonic distension thresholds in response to colonic distension were measured 30 min, 50 min, 70 min, and 120 min after dosing.

### 4.4. Assessment of Colonic Hypersensitivity in the Model of Partial Restraint Stress in Female Rats

Animals were placed for 2 h in a plexiglas restraint cylinder in order to restrict, but not impede, body movements. Naïve control animals were allowed to move freely in their cages. Immediately after completion of the 2 h restraint stress period, 3 consecutive determinations of colonic sensitivity were performed every 20 min. Vehicle (0.5% methylcellulose) or HC-070 were orally administered at 3 mL/kg, 50 min after the start of PRS, and visceral pain thresholds in response to colonic distension were measured 70 min, 90 min, and 110 min after dosing. Here, morphine was used as the positive analgesic control, as it is also clinically used in IBD patients, although it has reported effects on the visceral mechanical pain threshold [34]. It was subcutaneously administered, due to poor oral bioavailability, at 5 mL/kg, 90 min after the start of PRS, and visceral pain thresholds were assessed 30 min, 50 min, and 70 min after administration.

### 4.5. Measurement of the Visceral Pain Threshold Using the Colonic Distension Test in Rat TNBS and PRS Models

Colonic sensitivity was assessed by measuring the intra-colonic pressure required to induce a pain-related behavioral response during colonic distension (Electronic barostat, Distender Series IIRTM). Sedation is considered as a confounding factor in the assessment of pain, and therefore all experiments were performed in non-sedated conscious rats. In order to reduce bias, all experiments were performed by the same dedicated personnel within a study. The visceral pressure range was studied from 5 to 75 mmHg, but pain responses were typically evident at 30–35 mmHg. Briefly, a 5 cm balloon was gently inserted into the colon of conscious rats at 7 to 10 cm from the anus, and the catheter was taped to the base of the tail. After a 30 min acclimation period with the inserted balloon, colonic pressure was increased by 5 mmHg steps every 30 s, from 5 to 75 mmHg (cut-off), until pain-related elevation of the hind part of the rat body and/or a clearly visible abdominal contraction corresponding to severe cramp were evident.

### 4.6. Assessment of Mechanical Hypersensitivity Using the Paw Pressure or Randall and Selitto Test in the Model of Peripheral Mononeuropathy in Male Rats

Prior to behavioral studies, the rats were habituated to the experimental conditions and handling. The common sciatic nerve of the right hindpaw was injured at the level of the middle of the thigh. Proximal to the sciatic trifurcation, four loose ligatures were tied around it with about 1 mm spacing using Mersilk sutures in anaesthetized rats (Xylazine 10 mg/kg i.p., Ketamine 60 mg/kg i.p.). Great care was taken to tie the ligatures, such that the diameter of the nerve was seen to be just barely constricted. Sham animals received only a skin and muscle incision. Fourteen days after the surgery, individual baseline values of the withdrawal threshold evoked by squeezing of the hind limb between a flat surface and a blunt pointer with an analgesimeter (Ugo Basile, Gemonio, Italy) were determined on both hindpaws while the animal was gently held in a restrained position. The analgesimeter exerted a steadily increasing force, and the reaction threshold was determined as the pressure (g) required to elicit paw withdrawal and/or vocalization. Rats that met the inclusion criteria of 20 g < paw withdrawal threshold < 240 g for the injured paw, indicating static mechanical hyperalgesia-like hypersensitivity, and 280 g < paw withdrawal threshold < 520 g for the control paw, were then orally treated with vehicle (0.5% methylcellulose) and HC-070 at 3 mL/kg, or subcutaneously with morphine at 5 mL/kg. The oral bioavailability of morphine is well-known to be variable, and therefore morphine was dosed s.c. in order to reduce the inter-individual variation in exposure. The anti-hypersensitivity effect of the compounds was studied on both hindpaws using the paw pressure test 45 min, 75 min, and 105 min after treatment. Dosing and testing were performed in a random order.

### 4.7. Pharmacokinetics and Bioanalytical

Oral doses of HC-070 in 0.5% methylcellulose (MC) in water were administered at 1 mg/mL, 3.3 mg/mL, and 10 mg/mL at a 3 mL/kg dose volume to yield final doses of 3, 10, and 30 mg/kg, respectively, in the fed state. In parallel with sciatic nerve-injured animals, satellite groups of male rats were orally administered with HC-070, and plasma and brain were collected 45 and 75 min after dosing for exposure analyses. Terminal samples for concentration analyses were also collected from HC-070-treated sciatic nerve-injured male animals at the end of the paw pressure test, and from female rats at the end of the last colonic distension test in the partial restraint stress (PRS) study. Briefly, total blood was collected by intra-cardiac puncture on anaesthetized (3% Isoflurane, 3 L/min) animals into pre-cooled K3EDTA tubes. Samples were gently mixed, placed on ice, and centrifuged at the latest within 30 min after collection at 1600× *g* or 1710× *g* for 10 min at 4 °C. At least 100–200 µL of plasma was gently collected and immediately frozen at −80 °C. After decapitation, the whole brain was collected, rinsed with fresh ice-cold 0.9% NaCl solution, and thereafter frozen in dry ice and stored in a polypropylene tube at −80 °C. Liquid chromatography-tandem mass spectroscopy (LC-MS/MS) was used to determine HC-070 concentrations in rat plasma and brain homogenate samples.

### 4.8. Plasma Protein Binding and Brain Homogenate Binding

Plasma protein binding studies were carried out in male Wistar Hannover rat plasma with heparin as the anticoagulant. All plasma samples were obtained from BioreclamationIVT (Baltimore, MD, USA). A Pierce Rapid Equilibrium Dialysis Device (RED) was used for the experiments. Aliquots of plasma (300 µL) containing the compound of interest (1 µg/mL) and phosphate-buffered saline (PBS) (500 µL) were added to the sample chamber and buffer chamber, respectively, on the RED device. The loaded dialysis plate was sealed and placed on a pre-heated shaker, and incubated at physiological temperature (+37 °C) for 4 h. After the incubation, aliquots of 50 µL for the donor and 200 µL for the receiver were removed from the chambers and placed into a 96-well plate. Plasma (50 µL) was added to the wells containing the receiver samples and 200 µL of PBS was added to the wells containing the donor samples. Two volumes of ACN were added to each well, and the samples were mixed and centrifuged, after which the supernatant was analysed by LC-MS/MS.

The brain homogenate binding study was carried out in male Wistar Hannover brain homogenate, obtained from BioreclamationIVT (Baltimore, MD, USA). Brains were homogenized with two volumes of PBS prior to initiating the experiment. The instrumentation, volume ratios, incubation conditions, and sample preparation were the same as described above for plasma protein binding (brain homogenate used instead of plasma).

### 4.9. Drugs

HC-070 was synthesized by Orion Pharma (Espoo, Finland) and used as a TRPC4/5 channel antagonist at an oral dose range of 3–10 mg/kg in rat neuropathic and visceral pain models. HC-070 was dissolved in a suspension of 0.5% (*w/v*) methyl cellulose (MC) in purified water. Morphine was purchased from Francopia (Catalog reference 3695, Batch No. KR00017 or JR00002, Paris, France), dissolved in 0.9% NaCl solution, and used at a subcutaneous (s.c.) dose of 3 mg/kg in the rat neuropathic pain model or a dose of 1 mg/kg in the model of partial restraint stress in female rats. Trinitrobenzene sulfonic acid (TNBS), purchased from Sigma-Aldrich (Catalog reference 92822, Batch No. BCBT6864, Chesnes, France), solution (1 mL/kg) was prepared in 25% Ethanol Absolut. The κ-opioid agonist (−)U-50,488H, as the positive reference, was purchased from Sigma-Aldrich (Catalog reference U111, Batch No. 070M4626V), dissolved in 0.9% NaCl solution, and used at a subcutaneous (s.c.) dose of 3 mg/kg in the rat TNBS model.

### 4.10. Statistics

The formula: ((Distension threshold_TNBS/PRS + veh_ − Distension threshold_naïve + veh_)/(Distension threshold_naïve + veh_)) × 100, was used to calculate percentual variation due to trinitrobenzene sulfonic acid administration or partial restraint stress, as compared to naïve rats. The formula: ((Distension threshold_treated_ − Distension threshold_TNBS/PRS + veh_)/(Distension threshold_naïve + veh_ − Distension threshold_TNBS/PRS + veh_)) × 100, was used to illustrate the percentual magnitude of colonic anti-hypersensitivity effects for HC-070 and reference compounds in rat visceral pain models. Percentual reversal of CCI-induced sustained mechanical hypersensitivity by HC-070 and morphine was evaluated using the formula: ((Mechanical withdrawal threshold_treated_ − Mechanical withdrawal threshold_CCI_)/(Mechanical withdrawal threshold_control paw_ − Mechanical withdrawal threshold_CCI_) × 100). When results were normally distributed, data were analysed by a parametrical one-way or two-way repeated measures analysis of variance, followed by a Dunnett-corrected multiple comparison’s test to determine statistical significance for the effect of HC-070 and reference substances versus the vehicle, or for treatment and CCI-induced effects within groups. When results were not normally distributed, data were analyzed by a non-parametrical Kruskal–Wallis test, followed by a Dunn-corrected multiple comparison’s test to determine the statistical effects of HC-070 and reference substances, or by the Wilcoxon matched-pairs signed rank test to determine CCI-induced effects within groups. Statistical analyses were conducted using GraphPad Prism (version 8.4.2) and *p* < 0.05 was considered to represent a significant difference.

## 5. Conclusions

The present results indicated that efficacy with HC-070 in visceral and neuropathic pain models coincides with unbound brain concentrations near the 50% inhibitory concentration (IC_50_) for TRPC4 and suggest that central TRPC4/C5 channel inhibition is needed for analgesia. Moreover, HC-070 treatment did not have an impact on naive mechanical sensitivity. Taken together, these data support the hypothesis that TRPC4/C5 represent novel and safe non-opioid targets for the treatment of several pain conditions, in addition to attenuating suffering, depression, and anxiety associated with chronic pain.

## Data Availability

Data will be made available upon request.
